# Vitamin D supplementation for the prevention of type 2 diabetes in overweight adults: study protocol for a randomized controlled trial

**DOI:** 10.1186/s13063-015-0851-6

**Published:** 2015-08-07

**Authors:** Barbora de Courten, Aya Mousa, Negar Naderpoor, Helena Teede, Maximilian P J de Courten, Robert Scragg

**Affiliations:** Monash Centre for Health Research and Implementation, School of Public Health and Preventive Medicine, Monash University, 43-51 Kanooka Grove, Clayton, VIC 3186 Australia; Diabetes and Vascular Medicine Unit, Monash Health, Melbourne, Australia; Centre for Chronic Disease, Victoria University, Melbourne, Australia; School of Population Health, University of Auckland, Auckland, New Zealand

**Keywords:** 25(OH)D, inflammation, insulin secretion, insulin sensitivity, type 2 diabetes, vitamin D

## Abstract

**Background:**

Despite Australia’s sunny climate, low vitamin D levels are increasingly prevalent. Sun exposure is limited by long working hours, an increase in time spent indoors, and sun protection practices, and there is limited dietary vitamin D fortification. While the importance of vitamin D for bone mineralization is well known, its role as a protective agent against chronic diseases, such as type 2 diabetes and cardiovascular disease, is less understood. Observational and limited intervention studies suggest that vitamin D might improve insulin sensitivity and secretion, mainly via its anti-inflammatory properties, thereby decreasing the risk of development and progression of type 2 diabetes. The primary aim of this trial is to investigate whether improved plasma concentrations of 25-hydroxyvitamin D (25(OH)D), obtained through vitamin D supplementation, will increase insulin sensitivity and insulin secretion. A secondary aim is to determine whether these relationships are mediated by a reduction in underlying subclinical inflammation associated with obesity.

**Methods/Design:**

Fifty overweight but otherwise healthy nondiabetic adults between 18 and 60 years old, with low vitamin D levels (25(OH)D < 50 nmol/l), will be randomly assigned to intervention or placebo. At baseline, participants will undergo a medical review and anthropometric measurements, including dual X-ray absorptiometry, an intravenous glucose tolerance test, muscle and fat biopsies, a hyperinsulinemic euglycemic clamp, and questionnaires assessing diet, physical activity, sun exposure, back and knee pain, and depression. The intervention group will receive a first dose of 100,000 IU followed by 4,000 IU vitamin D (cholecalciferol) daily, while the placebo group will receive apparently identical capsules, both for a period of 16 weeks. All measurements will be repeated at follow-up, with the primary outcome measure expressed as a change from baseline in insulin sensitivity and secretion for the intervention group compared with the placebo group. Secondary outcome measures will compare changes in anthropometry, cardiovascular risk factors, and inflammatory markers.

**Discussion:**

The trial will provide much needed clinical evidence on the impact of vitamin D supplementation on insulin resistance and secretion and its underlying mechanisms, which are relevant for the prevention and management of type 2 diabetes.

**Trial registration:**

Clinicaltrials.gov ID: NCT02112721.

## Background

Vitamin D has well established endocrine functions in calcium absorption and healthy mineralization of bone as well as muscle function [1]. Low vitamin D levels have also been associated with many chronic diseases, including obesity [2], type 2 diabetes [3], cardiovascular disease [4], and cardiovascular and all-cause mortality [5]. However, knowledge gaps around vitamin D in these chronic diseases remain significant. This is increasingly clinically relevant today with vitamin D deficiency being prevalent worldwide as people adopt sedentary indoor lifestyles and use sunscreen and protective clothing to reduce the risk of skin cancer [1]. Diet is also not an ideal way to obtain vitamin D because few foods are naturally high in vitamin D and foods fortified with vitamin D are limited internationally [1]. The use of supplements offers a practical option for treating deficiency, while avoiding conflict with current public health measures for sun avoidance to protect against skin cancer [6]. However, the level of supplementation appropriate to improve vitamin D status remains controversial [7]. Currently, recommended daily oral intake is between 5 and 15 μg/day (200–600 IU/day) for adults (ages 19–70) [8, 9]. However, several recent studies suggested that an oral intake of at least 100 μg/day (4,000 IU/day) is required to raise serum concentrations of 25(OH)D to an optimal level within 2 to 3 months [10, 11]. Yet few existing studies have reached this level of replacement.

In relation to type 2 diabetes and cardiovascular risk factors, cross-sectional studies have shown that low plasma concentrations of 25(OH)D are associated with higher fasting serum glucose concentrations [12]; increased insulin resistance [13]; increased first and second phase insulin secretion [14, 15]; higher levels of haemoglobin A_1_c (HbA_1_c) [16, 17]; higher blood pressure [18, 19]; and higher levels of triglycerides and lower high-density lipoprotein cholesterol [20]. Prospectively, low serum concentrations of 25(OH)D have been associated with the development of insulin resistance [21], type 2 diabetes [22], hypertension [23], incidence of cardiovascular disease, and mortality [23–25]. Despite epidemiological data, there are relatively few good-quality interventional studies. Recent meta-analyses show significant heterogeneity in study quality, risk of bias, duration, dosages, and vitamin D deficiency status of participants, sample sizes, as well as surrogate measures of diabetes and cardiovascular disease, making it difficult to interpret study findings [23, 26]. In addition, many studies did not account for lifestyle factors, nor have they included dietary intake of vitamin D or calcium in their analyses, all of which affect both vitamin D levels and risk of type 2 diabetes and cardiovascular disease [23]. While recent evidence is limited and inconclusive, it does suggest that vitamin D might only have beneficial effects on vitamin D deficient individuals (25(OH)D < 50 nmol/l) who have received supplementation of sufficient dosages and durations, factors which require consideration in the design and execution of future trials.

Chronic low-grade inflammation is another factor that occurs in obesity. It is a risk factor for insulin resistance and many chronic diseases including type 2 diabetes and cardiovascular disease [27, 28]. There is evidence from *in-vitro* studies that vitamin D has anti-inflammatory properties [29–31]. However, there is a lack of human data [29].

While some large-scale trials are currently underway to assess the relationship between vitamin D and cardiovascular risk or disease [32], the role of vitamin D in insulin resistance or secretion and in regulating underlying chronic inflammation still needs to be assessed with good-quality trials and using gold-standard measures of glucose metabolism to address existing knowledge gaps [33].

To this end, we aim to perform a well-designed randomized placebo-controlled trial in healthy but overweight and obese individuals, as they are, on average, insulin resistant and have chronic low-grade inflammation. We aim to measure insulin sensitivity and secretion using gold-standard methodology, rather than examining glucose levels, HbA_1_c or using indirect measures of insulin sensitivity or secretion, which tend to change only slightly in nondiabetic individuals [23]. We will also measure blood pressure and lipid profile as surrogate measures of cardiovascular risk. Finally, chronic low-grade inflammation will be explored as a mechanism potentially linking low vitamin D levels and insulin sensitivity or secretion as well as cardiovascular risk factors.

## Methods/Design

### Study design and setting

**Fig. 1:**
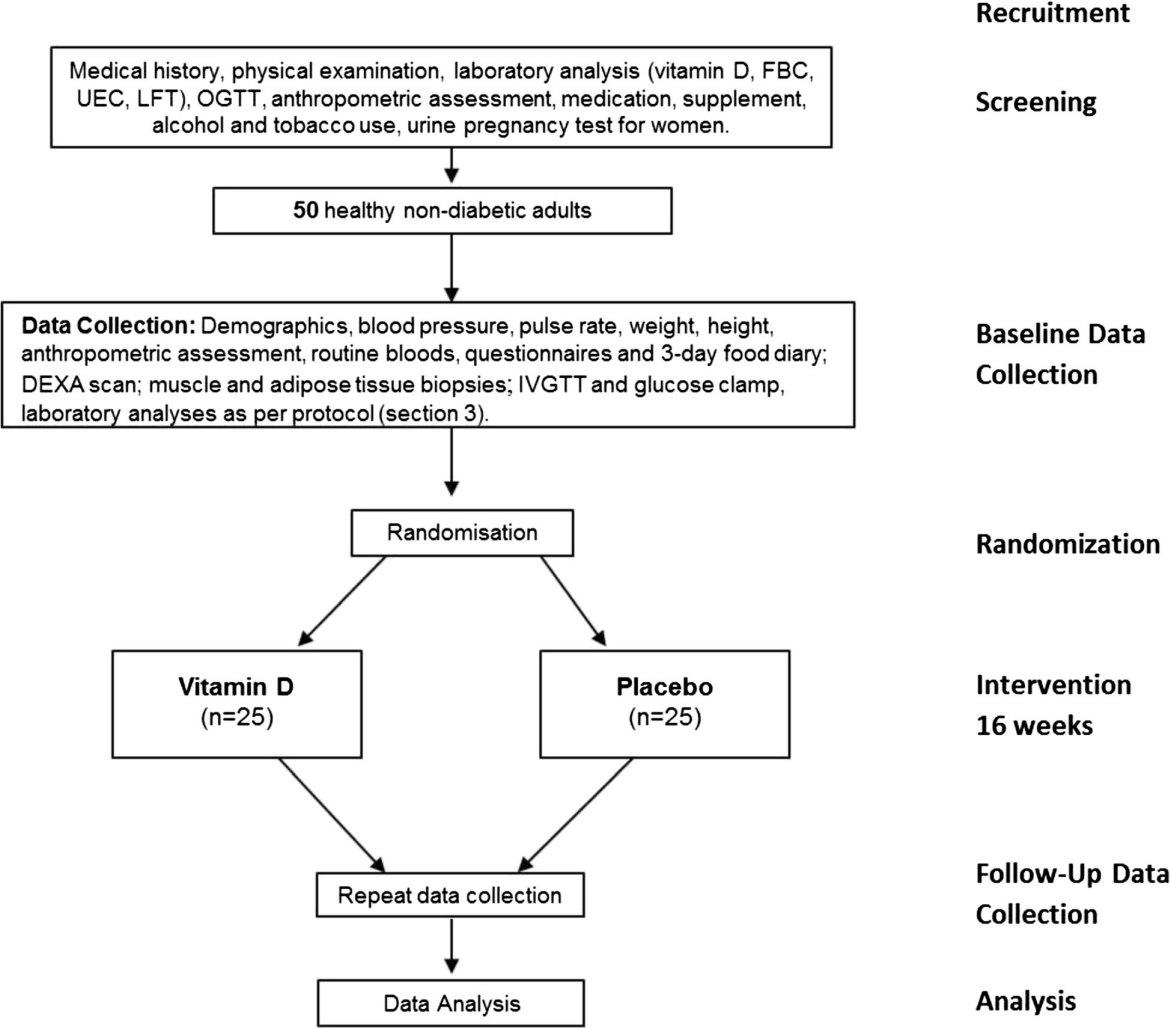
Trial protocol. DEXA, dual X-ray absorptiometry; FBC, full blood count; IVGTT, intravenous glucose tolerance test; LFT, liver function test; OGTT, oral glucose tolerance test; UEC, urea, electrolytes, creatinine

This study is a parallel-group randomized placebo-controlled trial. The study design is presented in Fig. 1. Fifty overweight but otherwise healthy normoglycaemic adults aged between 18 and 60 years, and with serum concentrations of 25(OH)D between 25 and 50 nmol/l on screening will be enrolled. Participants will be randomized to receive either placebo or vitamin D supplementation (25 participants in each arm) for 16 weeks, with a goal of achieving optimal 25(OH)D levels of approximately 100 nmol/l. Those with severe deficiency (serum 25(OH)D levels below 25 nmol/l) will not be included in the current trial but will be allocated to a nonrandomized mandatory treatment group and studied before and after supplementation. Overweight and obese subjects will be selected for this study, as they are generally more insulin resistant and more sedentary, and have higher levels of inflammatory markers and lower levels of vitamin D, all of which are factors that increase the risk of type 2 diabetes [34, 35]. Participants will be sought out via several advertising strategies, including posters, flyers, newspapers, and email newsletters at Monash University and Monash Medical Centre in Melbourne, Australia, and via online advertising avenues, including social media and popular community websites. Interested participants will be informed that their participation is voluntary and that they can withdraw at any time, and they will also be asked to read the details of the study provided in an information pack. Once all concerns and questions have been addressed, participants will be asked to sign an informed consent form prior to commencing any tests.

### Inclusion criteria

To take part in the trial, participants must be within the age range of 18 to 60 years inclusive, nondiabetic, and generally healthy, as determined by medical screening. Eligible participants must be overweight or obese (body mass index > 25 kg/m^2^). Their weight should not exceed 159 kg, owing to facility restrictions, and it should be stable, with a change of less than 5 kg in the preceding year. They should also have no intention to lose weight or change their diet or physical activity during the trial. To be included, individuals should also have a serum concentration of 25(OH)D of <50 nmol/l. However, those with frank vitamin D deficiency (<25 nmol/l) will not be randomized, but will instead be purposely allocated to the vitamin D group as allocation to placebo for 16 weeks might not be safe for these individuals.

### Exclusion criteria

Exclusion criteria will include smoking, high alcohol use (more than four standard drinks per week for men and more than two standard drinks per week for women), hypercalcemia, allergies, diabetes (previously diagnosed or on the basis of an oral glucose tolerance test), and the use of medication, including vitamins and other supplements. Based on medical history or physical or laboratory examination, participants will also be excluded if they have any kidney, cardiovascular, hematological, respiratory, gastrointestinal, endocrine or central nervous system diseases, as well as psychiatric disorders, active cancer within the preceding 5 years, or the presence of acute inflammation. Women who are experiencing menopause, pregnant, or lactating will also be excluded.

### Sample size calculation

Based on data from a similar healthy cohort of overweight or obese subjects in our metabolic laboratory, with a mean (standard deviation) insulin-mediated glucose uptake value of 8.1 (2.0) mg glucose per kg per minute, a sample size of 25 completing each arm is required to detect a 20 % change in insulin-mediated glucose uptake between treatment group and placebo. The 20 % increase is based on results from a study indicating the effect of vitamin D supplementation in subjects with diabetes using a comparable insulin sensitivity measurement technique and 4 weeks’ treatment [36]. Based on a type I error of 0.05 (two-tail) and a type II error of 0.20 (power = 80 %), we would require 50 participants to complete the trial.

### Screening

**Fig. 2:**
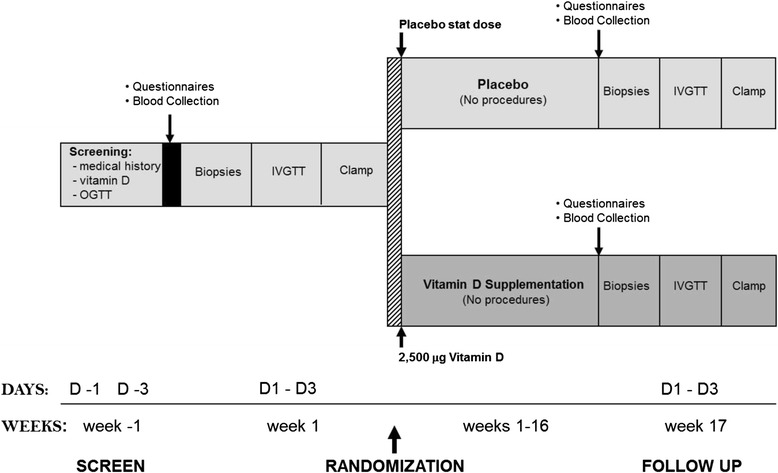
Study timeline. IVGTT, intravenous glucose tolerance test; OGTT, oral glucose tolerance test

The study timeline is presented in Fig. 2. Female participants who are not taking contraceptives will be required to commence metabolic testing in the follicular phase and a urine pregnancy test will be performed to exclude pregnancy. At visit 1, a signed informed consent form will be obtained from each participant followed by screening by a registered medical practitioner, who will collect medical history and perform a physical examination, including measurement of blood pressure, height, body weight, and waist and hip circumference.

At the second visit, participants will undergo an oral glucose tolerance test with measurement of fasting and 2 hour glucose levels to exclude diabetes according to World Health Organization guidelines [37]. Blood samples will be sent to Monash Pathology services to be analyzed for serum concentration of 25(OH)D as well as full blood count, kidney and liver function tests, lipid profile, parathyroid hormone levels, and concentration of calcium, magnesium, phosphate, and C-reactive protein (CRP) as a marker of inflammation.

### Baseline assessment

Baseline assessments will commence at the third visit, which will involve a muscle biopsy from the vastus lateralis, adipose tissue biopsies from subcutaneous abdominal tissue, and body composition assessment by dual X-ray absorptiometry (Monash Medical Centre Radiology Department). The final baseline visit will involve an intravenous glucose tolerance test and a hyperinsulinemic euglycemic clamp. Participants will complete validated questionnaires to assess self-reported sun exposure habits, physical activity (International Physical Activity Questionnaire) [38], knee and back pain, depression (Beck’s Depression Inventory) [39], and diet (3-day food record).

### Randomization

Following successful screening and baseline assessment, subjects will be randomized by a computerized randomization program to commence treatment with either vitamin D or placebo. This randomization will be done in blocks of four by sex and time of study entry (seasons) to ensure balance between the sexes in each test group and to control for the effect of seasonal change. Randomization of participants to the intervention or placebo groups will be completed by a researcher within our department (Monash Centre for Health Research and Implementation) who is not involved in the trial data collection, analysis, or reporting. This researcher will receive packaged supplements, produced by a clinical trials pharmacy, at Alfred Hospital. Capsules of vitamin D and placebo will be provided in clear containers and they will be identical and tasteless, to ensure that both participants and investigators are blinded to the treatment. To maximize compliance, participants will be provided with a calendar sheet highlighting the study period, so they can cross out each day as they go, and return the sheet along with the empty containers at the end of the study.

### Intervention

Following randomization, each participant will be given an initial first dose of 2,500 μg (100,000 IU) of vitamin D or an equivalent number of placebo tablets to be taken orally in front of researchers in the clinic. For those in the intervention group, this first dose is expected to elevate their serum concentration of 25(OH)D to 100 nmol/l within one week [10]. After the first dose, participants will take 100 μg/day (4,000 IU, four tablets) of vitamin D daily to keep their serum concentration of 25(OH)D at an optimal level. The daily dose selected is based on the amount required for repletion within a 3-month period [11], and is well below levels of intake associated with toxicity [40]. Participants on placebo will take four tablets daily of identical appearance. All participants will be instructed to consume four tablets daily, and otherwise maintain their usual daily activities.

### Follow-up visits

Participants will be scheduled for their follow-up visits during the last week of their treatment intake. All procedures performed during baseline assessment including blood pressure measurement, anthropometry, percutaneous muscle and adipose tissue biopsies, intravenous glucose tolerance testing, and glucose clamp will be repeated, except for the oral glucose tolerance test, as this is only performed initially to screen for diabetes. Participants will again be asked to fill out the same questionnaires and another 3-day food diary.

### Safety considerations

During the screening, baseline, and follow-up procedures, any medical conditions or abnormalities detected (except vitamin D status) will be promptly discussed with the participant by a qualified medical practitioner involved in the study. Where applicable, participants will be treated, referred, or advised to visit their general practitioner for follow-up. Individuals with frank vitamin D deficiency (<25 nmol/l) will not be included in the trial, as not treating these individuals would be unethical; hence, immediate treatment with vitamin D takes precedence. This severely deficient group will receive the same vitamin D supplementation and protocol (except randomization), and, along with their investigators, will remain blinded to maintain allocation concealment for this group. Results from these individuals will be analyzed separately from the trial. All participants will be informed of their screening blood test results after they have completed participation in the study, and they will be advised to visit their general practitioner to discuss strategies to increase or maintain their vitamin D levels, as well as improve their diabetes and cardiovascular risk profile.

### Ethics

This trial has received ethical approval from the Monash University Human Research Ethics Committee and Monash Health (protocol ID: CF13/3874 – 2013001988).

### Outcome measurements

The primary outcome measure in this trial is the difference (change) in insulin sensitivity between the vitamin D and placebo groups. Secondary outcome measures include changes in insulin secretion, weight, body mass index, waist, waist to hip ratio, resting systolic and diastolic blood pressure, lipid profile, and markers of inflammation, including IL-1β, IL-6, IL-8 and IL-10, CRP, TNFα, macrophage migration inhibitory factor, monocyte chemotactic protein-1, and NFκB activity. Measurement of insulin signaling in skeletal muscle and gene expression of inflammatory markers in adipose tissue will also be included as secondary outcome measures. Other outcome measures will include self-reported questionnaire data and nutrient analyses derived from dietary records.

### Data collection and analysis

#### Anthropometry

##### Body mass index

Using a digital scale (Tanita BWB-600, Australia) and stable stadiometer (Seca 206, Australia), body weight (kg) and height (m) will be measured, both at baseline and following the intervention period, during which participants will be lightly clothed and without shoes. These measurements will be converted to body mass index using the formula:

$$ \mathrm{Body}\ \mathrm{mass}\ \mathrm{index} = \mathrm{Weight}\ \left(\mathrm{kg}\right)\ /\ \mathrm{Height}\ {\left(\mathrm{m}\right)}^2. $$

##### Waist to hip ratio

Central adiposity will be assessed in duplicate using a constant-tension tape for measuring waist and hip circumference. Waist circumference will be measured by a single experienced researcher at the midpoint between the upper iliac crest and the lowermost rib at the end of a normal expiration, while hip circumference will be determined at around the widest part of the buttocks. Waist to hip ratio will be calculated using the formula:

$$ \mathrm{Waist}\ \mathrm{t}\mathrm{o}\ \mathrm{hip}\ \mathrm{ratio} = \mathrm{Waist}\ \mathrm{circumference}\ \left(\mathrm{cm}\right)\ /\ \mathrm{Hip}\ \mathrm{circumference}\ \left(\mathrm{cm}\right). $$

##### Body fat composition (dual X-ray absorptiometry)

Dual energy X-ray absorptiometry will be used to measure whole body fat composition. The fat tissue mass measured in this way consists of the sum of the fatty elements of all the soft tissue, not just adipose tissue, while the lean tissue mass reflects the sum of all chemical fat-free tissue components. Dual X-ray absorptiometry has been validated as an accurate, safe, and noninvasive method of measuring soft tissue body composition by region [41].

#### Metabolic measures

##### Oral glucose tolerance test

After a 10–12 hour overnight fast, participants will ingest 75 g of glucose over 2 min. Blood samples will be drawn at 0 and 120 minutes to analyze plasma glucose levels and determine diabetes status.

##### Percutaneous muscle and adipose tissue biopsy

Approximately 120 mg of skeletal muscle (vastus lateralis muscle) will be obtained by percutaneous muscle biopsy under local anaesthetic, immediately frozen and stored at −80 °C for later analysis. Adipose tissue (approximately 10 g) will be obtained by needle biopsy from the abdominal area, also under local anaesthetic, and will be immediately frozen and stored at −80 °C for later analysis.

##### Hyperinsulinemic euglycemic clamp

A euglycemic glucose clamp will be used to measure insulin sensitivity. After collecting baseline blood and plasma glucose levels at 0 min, the clamp will be initiated by an intravenous bolus injection of insulin (9 mU/kg). Insulin will then be constantly infused at a rate of 40 mU/(m^2^ min) for approximately 120 min into an arm vein, while glucose is variably infused to maintain euglycemia. Plasma glucose values will be monitored every 5 minutes during the clamp and the variable infusion rate of glucose will be adjusted to maintain blood glucose at a constant value of 5 mmol/l for at least 30 minutes.

##### Intravenous glucose tolerance test

Acute insulin secretory response will be measured using an intravenous glucose tolerance test. First, baseline blood will be collected at −10 and 0 min, after which 50 ml of 50 % glucose will be delivered intravenously over a 3 min period. Blood will then be collected for insulin concentration measurement at 3, 4, 5, 6, 8, 10, 15, 20, 25, and 30 min and glucose concentrations will be analyzed at each of these times, to determine insulin secretory response. This will be calculated as the average incremental plasma insulin level from the third to the fifth minute after the glucose bolus.

#### Cardiovascular measures

##### Blood pressure

Resting systolic and diastolic blood pressure and pulse rate will be measured using an automated oscillometric measurement system (Omron, Australia) after a 20-min rest. The average blood pressure derived from three measurements will be recorded.

##### Lipid profile

Lipid profile-related parameters to be measured include plasma total cholesterol, triglycerides, low-density and high-density lipoprotein cholesterol using a standard commercial enzymatic assay, a Beckman Coulter LX20PRO analyzer and SYNCHRON Systems lipid and multi calibrators (Beckman Coulter Diagnostics, Australia).

#### Inflammatory measures

##### Inflammatory markers

Plasma inflammatory markers (IL-1β, IL-6, IL-8 and IL-10, TNFα, macrophage migration inhibitory factor, and monocyte chemotactic protein-1) will be measured using a commercial automated chemiluminescent enzyme immunoassay analyzer (Immulite, Diagnostic Products Corporation, CA, USA), while plasma CRP will be analyzed via highly sensitive near infrared particle immunoassay rate methodology and a Beckman Coulter SYNCHRON LX system chemistry analyzer (Beckman Coulter Inc., Australia).

##### NFκB and Jun N-terminal protein kinase-1 activity

Nuclear extracts of white blood cells will be obtained and analyzed for the p50/p65 subunit of NFκB for their binding capacity for an NFκB consensus sequence (Active Motif, CA, USA). The bound subunits are sequentially incubated with specific antibodies to either the p50 or p65 subunit and then detected by their supershift on gel electrophoresis (Perkin Elmer, MA, USA). Activation of Jun N-terminal protein kinase-1 in the muscle and adipose tissue will be detected by immunochemistry and Western blotting, using antibodies from Cell Signaling Technology (MA, USA) and quantified using Quantity One imaging software (Bio-Rad Laboratories Inc., CA, USA).

#### Insulin signaling

We will measure changes in the expression and activation of important insulin signaling proteins, including the insulin receptor, insulin receptor substrate (IRS)-1 and 2, p85 subunit of phosphatidylinositol 3-kinase, and PKB/Akt, using Western blotting analysis, immunoprecipitation techniques, and commercially available phosphospecific antibodies (Santa Cruz Biotechnology, USA; Upstate Biotechnology, USA; Cell Signaling, MA, USA).

#### Gene expression

Gene expression of TNFα, IL-6, CRP, NFκB, and IKKβ in muscle and adipose tissue will be determined by real-time quantitative RT-PCR using the TaqMan system, based on real-time detection of accumulated fluorescence (ABI Prism 7700 sequence detection system; PE Biosystems, CA, USA). The protein concentration of all protein isolates will be determined using the bicinchoninic acid protein assay (Pierce Biotechnology Inc., IL, USA), according to the manufacturer’s instructions.

#### Other blood and tissue analyses

Other analyses include measurement of 25(OH)D concentration by direct competitive chemiluminescent immunoassay and a DiaSorin LIAISON Analyzer (DiaSorin Inc., USA) (CV 15 %); measurement of intact parathyroid hormone levels by an Access/DXI PTH assay, which is a paramagnetic particle chemiluminescent immunoassay for quantitative analysis (Beckman Coulter Inc., Australia); and measurement of insulin levels by Access/DXI ultrasensitive insulin assay, which is a simultaneous one-step immuno-enzymatic sandwich assay with CV levels of 7 %, 6.2 %, and 4.6 %, respectively (Beckman Coulter Inc., Australia). Serum concentrations of calcium, magnesium, and phosphate will be determined using automated colorimetric assays carried out on SYNCHRON LX and SYNCHRON DXC800® systems (Beckman Coulter Diagnostics, Australia), while plasma concentration of glucose will be determined using the SYNCHRON DXC800 and by an oxygen consumption rate method using a glucose oxygen electrode (Beckman Coulter Inc., Australia).

#### Self-reported measures

##### Nutrient analysis

Because some diets might be richer in vitamin D content than others, it was considered important to assess dietary practices of the study participants. The participants will be asked not to change their dietary habits during the study, and to complete a 3-day food diary, which will provide a snapshot of the types of food consumed by the participants when they entered the study as well as at follow-up, to ensure no major change in diet. These 3-day records will be analyzed with Food Works Professional 2007 (Xyris Software, Qld), a nutrient analysis program based on Australian food composition tables. Where possible, Australian data on the vitamin D content of foods will be used in the analyses [42]. Where Australian data are unavailable, a vitamin D database that we have recently compiled from UK, US, and European food composition tables will be employed.

##### Sun exposure questionnaire

Skin colour, owing to different quantities and compositions of melanin, affects the cutaneous photochemical synthesis of vitamin D [43]. Similarly, season and latitude influence vitamin D production in the body [43]. To take these into account, we have included a sun exposure questionnaire. Skin characteristics will be measured by grading hair colour (ten-point visual scale), eye colour, and reaction to sun exposure (four-point scale: *never* to *always* burn) [44]. Participants will be asked to estimate their average hours of sun exposure on a usual working day and on a nonworking day, to indicate how often sunscreen was used and its sun protection factor, and to describe the degree of clothing worn when outside during the summer and winter seasons. The description of clothing worn will then be used to determine the fraction of body surface area exposed to sunlight [45] and to calculate a sun index [45]:

$$ \mathrm{Sun}\ \mathrm{index} = \mathrm{Hours}\ \mathrm{of}\ \mathrm{sun}\ \mathrm{exposure}\ \mathrm{per}\ \mathrm{week} \times \mathrm{Fraction}\ \mathrm{of}\ \mathrm{body}\ \mathrm{surface}\ \mathrm{area}\ \mathrm{exposed} $$

##### International Physical Activity Questionnaire

The aim of the validated International Physical Activity Questionnaire is to establish the kinds of physical activity people engage in during their average day [38]. We opted to include the short version to make it more timely and convenient for participants, given that its purpose is to determine any change in physical activity that could influence our study outcomes. This version refers to the preceding 7 days and asks participants to report the number of days, and of hours and minutes, spent on vigorous activity, such as aerobics, or moderate activity, such as carrying light loads [38]. It also asks about the number of days, and of hours and minutes, participants walked for at least 10 minutes and how much time they spent sitting for any activity (work, travel, leisure, and so on) over the preceding 7 days.

Secondary outcome information derived from this short survey might provide some insight into whether different activity levels might be correlated with different vitamin D levels. While it is unlikely that we would reach any solid conclusions from this particular dataset, it might serve as a potential starting point for further, more comprehensive research in future.

##### Beck’s Depression Inventory

Beck’s Depression Inventory (2nd edition) is one of the most commonly used and validated diagnostic tools for depression and for determining its severity in diverse population groups, including different ethnicities and age groups, and in those with and without histories of depressive disorders or psychological distress [46, 47]. The influence of vitamin D deficiency on the likelihood of reporting depressive symptoms is currently unknown [48, 49], thus we have chosen to employ this questionnaire as a reliable tool for examining this relationship at baseline, and to assess whether depressive symptom reporting might change following vitamin D supplementation.

In this questionnaire, participants will be asked to report the frequency by which they have experienced such feelings as sadness, discouragement, dissatisfaction, guilt, indecisiveness, irritation, and suicidal thoughts, over the past 7 days. There are 21 multiple choice items in total, each with four possible responses ranging from a score of 0 to 3 [39, 50]. The scores for all 21 items are tallied to reach a total score between 0 and 63, with higher scores indicating more severe levels of depression.

##### Knee and back pain questionnaire

There has been a fair amount of evidence showing an association between circulating 25(OH)D concentrations and certain bone health outcomes, including osteoporosis, rickets, falls and fractures, and osteoarthritis [51, 52, 30]. However, research is lacking in relation to vitamin D and self-reported musculoskeletal pain. As such, we have added the knee and back pain questionnaire in an attempt to establish whether vitamin D supplementation might improve scores on self-reported pain scales and therefore prove useful for the treatment of high-risk individuals, such as older people and overweight or obese individuals. This questionnaire will collect information from participants regarding the amount of pain they currently experience in their dominant knee during certain activities, such as walking or standing, the duration in which they have experienced back pain, and the intensity of any back pain encountered within the past 6 months.

#### Statistical analysis

All analyses will be conducted according to the intention to treat principle. Baseline patient characteristics will be assessed using Student’s *t* test for continuous variables and the chi-squared test for categorical variables, to check for any imbalance between the two groups. Distributional assumptions will be checked and continuous variables will be transformed if normality is violated. Baseline and within-group differences will be assessed using paired Student’s *t* tests for both primary and secondary outcome measures. The effects of the intervention on the outcomes (between-group differences) at 16 weeks will be analyzed using changes in outcome variables and linear regression. Here, the variable of interest at 16 weeks will be the outcome variable minus baseline variable, which will be additionally adjusted for baseline values. Using linear regression, pre-specified subgroups, such as age categories, sex, and body mass index will be assessed for effect modification on anthropometric, metabolic, and cardiovascular outcomes.

A separate analysis will be conducted for the severely vitamin D deficient group, who will undergo a nonrandomized mandatory treatment with vitamin D supplementation as per protocol. A within-subject analysis on primary and secondary outcome measures using paired *t* tests will be conducted on this group.

All analyses will be performed using Stata statistical software. All tests will be two-sided and *P* values of less than 0.05 will be considered statistically significant.

## Discussion

To the best of our knowledge, this is the first clinical trial supplementing a sufficient dose of vitamin D in vitamin D deficient individuals, employing gold-standard measures of insulin sensitivity and secretion, and comprehensively investigating mechanisms of action including chronic low-grade inflammation in human beings.

Insulin resistance is common in obesity and is a key pathogenic process underpinning type 2 diabetes. Interventions that reduce insulin resistance, including lifestyle, weight loss, and pharmacological therapies, prevent and treat type 2 diabetes [53]; however, to date they have failed to slow the increasing burden of insulin resistance and resultant type 2 diabetes. Type 2 diabetes currently affects approximately 1 million Australians and, if it continues to rise at the current rate, it is estimated that this number will increase to 3 million by 2025 [31]. Diabetes is a major cause of morbidity and mortality, primarily due to an increased risk of cardiovascular disease. Moreover, Australians with type 2 diabetes require healthcare, carers, and government subsidies totalling up to 6 billion dollars in healthcare costs annually. It is vital that additional effective primary prevention strategies are established to reduce insulin resistance and prevent and manage type 2 diabetes [31]. This trial will thus inform and advance this important field.

If vitamin D supplementation improves insulin sensitivity or secretion in this trial, then with large-scale interventions, treating vitamin D deficiency could become a mainstream strategy for diabetes prevention in overweight and obese individuals who are deficient in vitamin D. Vitamin D supplementation would offer a cost-effective and easily administered intervention that could have a considerable impact on health outcomes in Australia and worldwide. In addition, vitamin D could also play a role in counteracting adverse cardiovascular events, as well as improving underlying inflammatory responses, thereby decreasing risk factors associated with a range of metabolic conditions. High-quality clinical trials, such as this, are therefore an important first step towards elucidating the true potential of vitamin D supplementation in promoting health and well-being, and reducing insulin resistance and potentially the risk of type 2 diabetes and its associated comorbidities.

Recent meta-analyses of studies examining the effect of vitamin D on type 2 diabetes and cardiovascular disease risk factors have produced largely negative results [23, 26]. Yet, some studies have suggested that improvements are only observed in vitamin D deficient individuals and only with adequate vitamin D supplementation [24, 36, 54]. Nevertheless, few trials have involved vitamin D deficient participants or provided adequate dosages, highlighting the need for trials that address these knowledge gaps.

Mechanisms of action of vitamin D might include direct and indirect effects on both insulin sensitivity and secretion, which have been investigated largely in animal and *in-vitro* studies. Vitamin D was shown to increase transcriptional activation and expression of the insulin receptor gene, which facilitates both basal- and insulin-stimulated glucose oxidation and results in improved insulin sensitivity [55–57]. Vitamin D also enhances insulin action and signal transduction by regulating extracellular calcium [58]. Vitamin D influences β-cell insulin secretion by elevating intracellular calcium, which is a crucial mechanism involved in β-cell glycolysis and the signaling of circulating glucose [29]. Vitamin D has also been implicated in the modulation of cytokine mediated β-cell apoptosis, the most important factor in the development and progression of type 2 diabetes [59, 60]. Notably, vitamin D also counteracts the harmful effects of advanced glycation products, which are essential for the development of type 2 diabetes complications and have recently been associated with the development of insulin resistance [61]. The presence of vitamin D receptors and vitamin D binding protein in pancreatic islets and nearly all inflammatory cells supports the idea that vitamin D plays a crucial role in the function of these cells, and potentially in the subsequent development of type 2 diabetes [62]. However, high-quality randomized trials remain scarce, and thus the current clinical trial plans to address this gap by comprehensively investigating mechanisms of vitamin D action *in-vivo* in human beings.

The mechanism by which vitamin D is believed to improve cardiac function and reduce the risk of hypertension is primarily via downregulating the renin-angiotensin-aldosterone system [63–66]. Vitamin D reduces levels of angiotensin II in the plasma, thereby reducing angiotensin-II-induced vasoconstriction [63–65]. Consistent with this notion, those who are deficient in vitamin D were found to have heightened levels of circulating angiotensin II, thereby increasing their risk of hypertension [63]. Vitamin D also enhances endothelial vasodilatation and modulates the flux of calcium, leading to reduced secretion of renin by juxtaglomerular cells present in vascular smooth muscle tissue, which could explain some of the antihypertensive effects of vitamin D [63–65]. Vitamin D, through its effects on advanced glycation products and oxidative stress [67], might also alleviate hypertension and reduce the risk of cardiovascular disease [68]. Another link between vitamin D and cardiovascular health is through its direct and indirect effects on lipid profiles. The indirect mechanism again refers to the calcium-regulating function of vitamin D, whereby increased calcium levels are proposed to reduce hepatic triglyceride formation and secretion [20]. It has also been suggested that vitamin D receptors improve lipid metabolism by reducing acetylated low-density lipoprotein cholesterol uptake [69]. More direct effects include the promotion of high-density lipoprotein cholesterol particle formation and the regulation of serum apolipoprotein A-1 levels by vitamin D, both of which contribute to increased cholesterol transport and overall improved lipid profiles [69–71].

Research investigating the role of vitamin D in reducing underlying chronic inflammation has also been fairly limited. However, preliminary findings suggest multiple nongenomic actions for vitamin D, alongside observed alterations in gene and protein expression following vitamin D treatment [59, 72]. For example, *in-vitro* investigations have identified the presence of vitamin D receptors in dendritic cells, monocytes and activated lymphocytes, among other inflammatory cells [62]. Moreover, 1,25(OH)D_3_ applied to isolated monocytes has been shown to regulate chemokine and cytokine secretion in patients with type 2 diabetes [72]. This in turn inhibits the pro-inflammatory actions of monocytes and decreases the proliferation and stimulatory abilities of T-cells, both of which are mechanisms thought to be due to a reduction in the expression of surface markers including major histocompatibility complex-II [72]. Where the absence of vitamin D receptors was observed, there appeared to be an escalation of pro-inflammatory activity by such cytokines as TNF-α, IL6, and CRP, which is thought to result primarily from an increase in NFκB activity [73, 29]. Supporting this notion, studies have also found that NFκB translocation appeared to be arrested, and its activity diminished, by the active form of vitamin D [74–76]. From these limited *in-vitro* studies stems the current belief that vitamin D is, at least in part, involved in regulating the signaling and stimulation of NFκB, while also controlling both separate and subsequent cytokine activity. Nevertheless, research in human beings, particularly in clinical trials of vitamin D supplementation or investigating chronic inflammation, are lacking. This trial can therefore corroborate previous knowledge, while also providing important explanations regarding the role of vitamin D in human inflammatory responses, and subsequent development of disease.

### Methodological considerations

The strengths of the current study include the gold-standard study methodology, employing both randomization and double-blinding of both participants and investigators, to limit bias; the placebo-controlled design; the use of supplementary questionnaires to control for possible confounders, such as diet, physical activity, sun exposure, and seasonality; the involvement of vitamin D deficient participants; the use of an optimal dose of 4,000 IU vitamin D per day for 16 weeks, considered sufficient to raise plasma 25(OH)D concentrations; and the use of direct measures of insulin sensitivity and secretion, such as the oral glucose tolerance test, intravenous glucose tolerance test, and gold-standard euglycemic clamp, alongside comprehensive exploration of mechanisms of vitamin D action.

Despite these strengths, there are also potential limitations. First, there might be self-selection bias, given that recruitment is achieved via voluntary participation by interested subjects. These subjects might not represent the entire target population because they might be characteristically different from the volunteers (that is, potentially more health-conscious). However, randomization should serve as an effective means of minimizing this risk. Second, because we are only recruiting adults with a body mass index above 25 kg/m^2^, but who are otherwise healthy (nondiabetic, no medication, and so on), the results of the study would not be generalizable to other populations, such as those within a healthy weight range, children, or those with diagnosed diabetes or other medical conditions and comorbidities. Third, our study will only measure surrogate markers, such as blood pressure, fasting glucose, and insulin sensitivity and insulin secretion. Although these are risk factors for diabetes and cardiovascular disease, longitudinal follow-up studies would be necessary to ascertain whether a change in these risk factors translates into decreased incidence of diabetes and cardiovascular disease.

Finally, the self-report nature of the questionnaire components of this study might introduce social desirability bias, scale format and anchor biases, or other construct and formatting related problems [77]. However, most of the questionnaires selected have been validated for construct and internal validity, among other quality indicators. Further, the double-blind design will ensure any impact from these biases occurs equally in both groups. The questionnaire data are secondary outcome measures and will be used to supplement the objective measurements obtained in the study. Data from these sources are thus not treated as definitive findings, nor are they intended to establish any aspects of causality.

Vitamin D deficiency is now pandemic across most populations, but with the use of supplements, treatment can easily be administered on a population-wide level. Before this can occur, however, randomized trials, such as the one described here, are necessary, to elucidate the effect of vitamin D on chronic conditions, including type 2 diabetes and cardiovascular disease. If an effect is established in our clinical trial, it could serve as a basis for future research and lead to feasible and cost-effective strategies to treat vitamin D deficiency and decrease the risk of type 2 diabetes and cardiovascular disease on a global scale.

## Trial status

This randomized trial commenced in July 2014, with the first eligible participant enrolment taking place on 27 July 2014. The trial is currently ongoing and actively recruiting, and expected to take approximately 3 years (from the commencement date to completion).

## Abbreviations

25(OH)D: 25-hydroxy vitamin D
CRP: C-reactive protein
HbA_1_c: haemoglobin A_1_c
IL: interleukin
NFκB: nuclear factor κB
TNF-α: tumor necrosis factor-α

## References

[1] Holick MF (2009). Vitamin D, status: measurement, interpretation, and clinical application. Ann Epidemiol.

[2] Hypponen E, Power C (2007). Hypovitaminosis D in British adults at age 45 y: nationwide cohort study of dietary and lifestyle predictors. Am J Clin Nutr.

[3] Pittas A, Lau J, Hu F, Dawson-Hughes B (2007). The role of Vitamin D and calcium in type 2 diabetes: a systematic review and meta-analysis. J Clin Endocrinol Metab.

[4] Grandi NC, Breitling LP, Brenner H (2010). Vitamin D and cardiovascular disease: systematic review and meta-analysis of prospective studies. Prev Med.

[5] Joergensen C, Gall MA, Schmedes A, Tarnow L, Parving HH, Rossing P (2010). Vitamin D levels and mortality in type 2 diabetes. Diabetes Care.

[6] Sinclair C (2006). Risks and benefits of sun exposure: implications for public health practice based on the Australian experience. Prog Biophys Mol Biol.

[7] Holick MF (2011). Vitamin D, deficiency in 2010: health benefits of vitamin D and sunlight: a D-bate. Nat Rev Endocrinol.

[8] Government A (2006). Department of Health and Aging, National Health and Medical Research Council. Nutrient reference values for Australia and New Zealand: executive summary.

[9] Ross AC, Manson JE, Abrams SA, Aloia JF, Brannon PM, Clinton SK (2011). The 2011 report on dietary reference intakes for calcium and vitamin D from the Institute of Medicine: what clinicians need to know. J Clin Endocrinol Metab.

[10] Ilahi M, Armas LA, Heaney RP (2008). Pharmacokinetics of a single, large dose of cholecalciferol. Am J Clin Nutr.

[11] Vieth R, Chan PC, MacFarlane GD (2001). Efficacy and safety of vitamin D_3_ intake exceeding the lowest observed adverse effect level. Am J Clin Nutr.

[12] Need AG, O’Loughlin PD, Horowitz M, Nordin BE (2005). Relationship between fasting serum glucose, age, body mass index and serum 25 hydroxyvitamin D in postmenopausal women. Clin Endocrinol.

[13] Liu E, Meigs JB, Pittas AG, McKeown NM, Economos CD, Booth SL (2009). Plasma 25-hydroxyvitamin D is associated with markers of the insulin resistant phenotype in nondiabetic adults. J Nutr.

[14] Chiu KC, Chu A, Go VL, Saad MF (2004). Hypovitaminosis D is associated with insulin resistance and β cell dysfunction. Am J Clin Nutr.

[15] Ou HY, Karnchanasorn R, Lee LZ, Chiu KC (2011). Interaction of BMI with vitamin D and insulin sensitivity. Eur J Clin Investig.

[16] Hypponen E, Power C (2006). Vitamin D status and glucose homeostasis in the 1958 British birth cohort: the role of obesity. Diabetes Care.

[17] Pinelli NR, Jaber LA, Brown MB, Herman WH (2010). Serum 25-hydroxy vitamin D and insulin resistance, metabolic syndrome, and glucose intolerance among Arab Americans. Diabetes Care.

[18] Gannage-Yared MH, Chedid R, Khalife S, Azzi E, Zoghbi F, Halaby G (2009). Vitamin D in relation to metabolic risk factors, insulin sensitivity and adiponectin in a young Middle-Eastern population. Eur J Endocrinol.

[19] Burgaz A, Byberg L, Rautiainen S, Orsini N, Hakansson N, Arnlov J (2011). Confirmed hypertension and plasma 25(OH)D concentrations amongst elderly men. J Intern Med.

[20] Jorde R, Grimnes G (2011). Vitamin D and metabolic health with special reference to the effect of vitamin D on serum lipids. Prog Lipid Res.

[21] Forouhi NG, Luan J, Cooper A, Boucher BJ, Wareham NJ (2008). Baseline serum 25-hydroxy vitamin D is predictive of future glycemic status and insulin resistance: the Medical Research Council Ely Prospective Study 1990–2000. Diabetes.

[22] Gagnon C, Lu ZX, Magliano DJ, Dunstan DW, Shaw JE, Zimmet PZ (2011). Serum 25-hydroxyvitamin D, calcium intake, and risk of type 2 diabetes after 5 years: results from a national, population-based prospective study (the Australian Diabetes, Obesity and Lifestyle study). Diabetes Care.

[23] Pittas AG, Chung M, Trikalinos T, Mitri J, Brendel M, Patel K (2010). Systematic review: Vitamin D and cardiometabolic outcomes. Ann Intern Med.

[24] Zittermann A (2014). Vitamin D, and cardiovascular disease. Anticancer Res.

[25] Zittermann A, Prokop S (2014). The role of vitamin D for cardiovascular disease and overall mortality. Adv Exp Med Biol.

[26] Seida JC, Mitri J, Colmers IN, Majumdar SR, Davidson MB, Edwards AL (2014). Clinical review: effect of vitamin D_3_ supplementation on improving glucose homeostasis and preventing diabetes: a systematic review and meta-analysis. J Clin Endocrinol Metab.

[27] Vozarova B, Weyer C, Lindsay RS, Pratley RE, Bogardus C, Tataranni PA (2002). High white blood cell count is associated with a worsening of insulin sensitivity and predicts the development of type 2 diabetes. Diabetes.

[28] Eizirik DL, Cardozo AK, Cnop M (2008). The role for endoplasmic reticulum stress in diabetes mellitus. Endocr Rev.

[29] Palomer X, Gonzalez-Clemente JM, Blanco-Vaca F, Mauricio D (2008). Role of vitamin D in the pathogenesis of type 2 diabetes mellitus. Diabetes Obes Metab.

[30] Sanghi D, Mishra A, Sharma AC, Singh A, Natu SM, Agarwal S (2013). Does vitamin D improve osteoarthritis of the knee: a randomized controlled pilot trial. Clin Orthop Relat Res.

[31] Shaw J, Tanama S (2012). Diabetes: the silent pandemic and its impact on Australia.

[32] Kupferschmidt K (2012). Uncertain verdict as vitamin D goes on trial. Science.

[33] Mitri J, Muraru MD, Pittas AG (2011). Vitamin D and type 2 diabetes: a systematic review. Eur J Clin Nutr.

[34] Arunabh S, Pollack S, Yeh J, Aloia JF (2003). Body fat content and 25-hydroxyvitamin D levels in healthy women. J Clin Endocrinol Metab.

[35] Bischof MG, Heinze G, Vierhapper H (2006). Vitamin D status and its relation to age and body mass index. Horm Res.

[36] Borissova AM, Tankova T, Kirilov G, Dakovska L, Kovacheva R (2003). The effect of vitamin D_3_ on insulin secretion and peripheral insulin sensitivity in type 2 diabetic patients. Int J Clin Pract.

[37] Alberti KG, Zimmet PZ (1998). Definition, diagnosis and classification of diabetes mellitus and its complications. Part 1: diagnosis and classification of diabetes mellitus provisional report of a WHO consultation. Diabet Med.

[38] Craig CL, Marshall AL, Sjostrom M, Bauman AE, Booth ML, Ainsworth BE (2003). International physical activity questionnaire: 12-country reliability and validity. Med Sci Sports Exerc.

[39] Beck AT, Steer RA, Carbin MG (1988). Psychometric properties of the Beck Depression Inventory: twenty-five years of evaluation. Clin Psychol Rev.

[40] Hathcock JN, Shao A, Vieth R, Heaney R (2007). Risk assessment for vitamin D. Am J Clin Nutr.

[41] Svendsen OL, Haarbo J, Hassager C, Christiansen C (1993). Accuracy of measurements of body composition by dual-energy X-ray absorptiometry *in vivo*. Am J Clin Nutr.

[42] Food Standard Australia New Zealand. NUTTAB 2010. http://www.foodstandards.gov.au/Pages/default.aspx

[43] Norman AW (1998). Sunlight, season, skin pigmentation, vitamin D, and 25-hydroxyvitamin D: integral components of the vitamin D endocrine system. Am J Clin Nutr.

[44] Rosso S, Minarro R, Schraub S, Tumino R, Franceschi S, Zanetti R (2002). Reproduceability of skin characteristic measurements and reported sun exposure history. Int J Epidemiol.

[45] Barger-Lux MJ, Heaney RP (2002). Effects of above average summer sun exposure on serum 25-hydroxyvitamin D and calcium absorption. J Clin Endocrinol Metab.

[46] Carmody DP (2005). Psychometric characteristics of the Beck Depression Inventory-II with college students of diverse ethnicity. Int J Psychiatry Clin Pract.

[47] Schotte CK, Maes M, Cluydts R, De Doncker D, Cosyns P (1997). Construct validity of the Beck Depression Inventory in a depressive population. J Affect Disord.

[48] Chung HK, Cho Y, Choi S, Shin MJ (2014). The association between serum 25-hydroxyvitamin D concentrations and depressive symptoms in Korean adults: findings from the fifth Korea National Health and Nutrition Examination Survey 2010. PLoS One.

[49] Howland RH (2011). Vitamin D, and depression. J Psychosoc Nurs Ment Health Serv.

[50] Siegert RJ, Tennant A, Turner-Stokes L (2010). Rasch analysis of the Beck Depression Inventory-II in a neurological rehabilitation sample. Disabil Rehabil.

[51] Al-Jarallah K, Shehab D, Abraham M, Mojiminiyi OA, Abdella NA (2013). Musculoskeletal pain: should physicians test for vitamin D level?. Int J Rheum Dis.

[52] Cranney A, Horsley T, O’Donnell S, Weiler H, Puil L, Ooi D (2007). Effectiveness and safety of vitamin D in relation to bone health. Evid Rep Technol Assess.

[53] Hamman RF, Horton E, Barrett-Connor E, Bray GA, Christophi C, Crandall J (2015). Factors affecting the decline in incidence of diabetes in the Diabetes Prevention Program Outcome Study (DPPOS). Diabetes.

[54] von Hurst PR, Stonehouse W, Coad J (2010). Vitamin D supplementation reduces insulin resistance in South Asian women living in New Zealand who are insulin resistant and vitamin D deficient: a randomised, placebo-controlled trial. Br J Nutr.

[55] Dunlop TW, Vaisanen S, Frank C, Molnar F, Sinkkonen L, Carlberg C (2005). The human peroxisome proliferator-activated receptor δ gene is a primary target of 1α,25-dihydroxyvitamin D_3_ and its nuclear receptor. J Mol Biol.

[56] Maestro B, Campion J, Davila N, Calle C (2000). Stimulation by 1,25-dihydroxyvitamin D_3_ of insulin receptor expression and insulin responsiveness for glucose transport in U-937 human promonocytic cells. Endocr J.

[57] Maestro B, Molero S, Bajo S, Davila N, Calle C (2002). Transcriptional activation of the human insulin receptor gene by 1,25-dihydroxyvitamin D_3_. Cell Biochem Funct.

[58] Cavalier E, Delanaye P, Souberbielle JC, Radermecker RP (2011). Vitamin D and type 2 diabetes mellitus: where do we stand?. Diabetes Metab.

[59] Dutta D, Mondal SA, Choudhuri S, Maisnam I, Hasanoor Reza AH, Bhattacharya B (2014). Vitamin-D supplementation in prediabetes reduced progression to type 2 diabetes and was associated with decreased insulin resistance and systemic inflammation: an open label randomized prospective study from Eastern India. Diabetes Res Clin Pract.

[60] Pratley RE, Weyer C (2002). Progression from IGT to type 2 diabetes mellitus: the central role of impaired early insulin secretion. Curr Diab Rep.

[61] Mark AB, Poulsen MW, Andersen S, Andersen JM, Bak MJ, Ritz C (2014). Consumption of a diet low in advanced glycation end products for 4 weeks improves insulin sensitivity in overweight women. Diabetes Care.

[62] Veldman CM, Cantorna MT, DeLuca HF (2000). Expression of 1,25-dihydroxyvitamin D_3_ receptor in the immune system. Arch Biochem Biophys.

[63] Baz-Hecht M, Goldfine AB (2010). The impact of vitamin D deficiency on diabetes and cardiovascular risk. Curr Opin Endocrinol Diabetes Obes.

[64] Farmer JA (2000). Renin angiotensin system and ASCVD. Curr Opin Cardiol.

[65] Milliez P, Girerd X, Plouin PF, Blacher J, Safar ME, Mourad JJ (2005). Evidence for an increased rate of cardiovascular events in patients with primary aldosteronism. J Am Coll Cardiol.

[66] Ford JA, MacLennan GS, Avenell A, Bolland M, Grey A, Witham M (2014). Cardiovascular disease and vitamin D supplementation: trial analysis, systematic review, and meta-analysis. Am J Clin Nutr.

[67] Vaidya A, Forman JP (2012). Vitamin D and vascular disease: the current and future status of vitamin D therapy in hypertension and kidney disease. Curr Hypertens Rep.

[68] Sourris KC, Lyons JG, Dougherty SL, Chand V, Straznicky NE, Schlaich MP (2014). Plasma advanced glycation end products (AGEs) and NF-κB activity are independent determinants of diastolic and pulse pressure. Clin Chem Lab Med.

[69] Al-Daghri NM, Alkharfy KM, Al-Saleh Y, Al-Attas OS, Alokail MS, Al-Othman A (2012). Modest reversal of metabolic syndrome manifestations with vitamin D status correction: a 12-month prospective study. Metab Clin Exp.

[70] Carbone LD, Rosenberg EW, Tolley EA, Holick MF, Hughes TA, Watsky MA (2008). 25-Hydroxyvitamin D, cholesterol, and ultraviolet irradiation. Metab Clin Exp.

[71] Wehmeier KR, Mazza A, Hachem S, Ligaray K, Mooradian AD, Wong NC (2008). Differential regulation of apolipoprotein A-I gene expression by vitamin D receptor modulators. Biochim Biophys Acta.

[72] Wolden-Kirk H, Overbergh L, Christesen HT, Brusgaard K, Mathieu C (2011). Vitamin D and diabetes: its importance for beta cell and immune function. Mol Cell Endocrinol.

[73] Giulietti A, van Etten E, Overbergh L, Stoffels K, Bouillon R, Mathieu C (2007). Monocytes from type 2 diabetic patients have a pro-inflammatory profile. 1,25-dihydroxyvitamin D_3_ works as anti-inflammatory. Diabetes Res Clin Pract.

[74] Deb DK, Chen Y, Zhang Z, Zhang Y, Szeto FL, Wong KE (2009). 1,25-dihydroxyvitamin D_3_ suppresses high glucose-induced angiotensinogen expression in kidney cells by blocking the NF-κB pathway. Am J Physiol Renal Physiol.

[75] Harant H, Andrew PJ, Reddy GS, Foglar E, Lindley IJ (1997). 1α,25-dihydroxyvitamin D_3_ and a variety of its natural metabolites transcriptionally repress nuclear-factor-κB-mediated interleukin-8 gene expression. Eur J Biochem.

[76] Szeto FL, Sun J, Kong J, Duan Y, Liao A, Madara JL (2007). Involvement of the vitamin D receptor in the regulation of NF-κB activity in fibroblasts. J Steroid Biochem Mol Biol.

[77] Podsakoff PM, MacKenzie SB, Lee JY, Podsakoff NP (2003). Common method biases in behavioral research: a critical review of the literature and recommended remedies. J Appl Psychol.

